# CXCR Family and Hematologic Malignancies in the Bone Marrow Microenvironment

**DOI:** 10.3390/biom15050716

**Published:** 2025-05-13

**Authors:** Yanquan Liu, Huanwen Tang

**Affiliations:** 1 Department of Hematology, The First Dongguan Affiliated Hospital of Guangdong Medical University, The First School of Clinical Medicine, Guangdong Medical University, Dongguan 523808, China; doctorliuyanquan@gdmu.edu.cn; 2 Key Laboratory on Leukemia of Jiangxi Provincial Health Commission, Department of Hematology, The Affiliated Ganzhou Hospital of Nanchang University (Ganzhou People’s Hospital), Ganzhou 341000, China; 3 National Key Laboratory of Hematology, Fujian Institute of Hematology, Fujian Medical University Union Hospital, Fuzhou 350001, China; 4 Department of Hematology, The First Dongguan Affiliated Hospital of Guangdong Medical University, Dongguan Key Laboratory of Environmental Medicine, School of Public Health, Guangdong Medical University, Dongguan 523808, China

**Keywords:** CXC chemokine receptor, hematologic malignancies, lymphopoietic system, carcinogenic mechanisms, therapeutic targets

## Abstract

Malignant hematologic diseases, also referred to as hematologic tumors, encompass a series of malignant proliferative disorders of the lymphopoietic system, including leukemia, lymphoma, multiple myeloma, and myeloproliferative neoplasms. The dysregulation of inflammatory factors or chronic inflammatory responses plays an indispensable role in the onset and progression of these tumors. The C-X-C motif chemokine receptor (CXCR) serves as a key mediator of immune-inflammatory responses. Through its specific regulatory mechanisms, CXCR is involved in the transduction and activation of various signaling pathways, thereby mediating the malignant biological characteristics of blood tumor cells, such as uncontrolled proliferation, differentiation, invasion, migration, autophagy, and apoptosis. In the bone marrow microenvironment, CXCR plays a pivotal role. This review systematically analyzes and elucidates the roles and mechanisms of the CXCR family in hematologic malignancies, aiming to provide new insights into the biological mechanisms and clinical significance of these diseases. The CXCR family holds great potential as a molecular marker for both fundamental research and the clinical diagnosis and treatment of hematologic malignancies.

## 1. Introduction

As a class of non-solid malignant tumors originating from the bone marrow microenvironment and lymphohematopoietic system with high heterogeneity, malignant hematologic diseases encompass myelodysplastic syndrome (MDS), leukemia, lymphoma, multiple myeloma (MM), myeloproliferative neoplasm (MPN), and others. These diseases can occur at any age and are typically associated with poor prognosis, high recurrence rates, and mortality. They represent incurable malignancies that pose severe threats to human life and health, imposing a significant medical and economic burden and leading to social instability [1]. In recent years, the mechanisms involving the CXCR family in malignant hematological diseases have been progressively elucidated by domestic and international scholars, becoming a focal point in academic research.

The CXCR family belongs to the G protein-coupled receptor superfamily and plays a critical role in inducing inflammatory chemotaxis and regulating the malignant biological behavior of tumor cells through interactions with specific CXC chemokine ligands (CXCLs) [2]. It is worth noting that the bone marrow microenvironment (BME) is closely linked to cellular malignant transformation and provides fertile ground for tumor initiation, progression, and metastasis. However, our current understanding of the cellular composition, distribution, interactions, and regulatory networks of TME or BME remains limited. Chemokines and their receptors constitute a class of small secreted proteins (8–12 kDa) produced by stromal or tumor cells, exhibiting multifaceted effects such as activation, attraction, and recruitment. Based on the arrangement of N-terminal cysteine (C) residues, chemokine receptors are categorized into four classes: CXCR, CCR, CX3CR, and CR. Among these, CXCR represents one of the most significant chemokine receptors [3,4].

The CXCR family comprises CXCR1-8, predominantly expressed on the surfaces of immunoinflammatory cells (e.g., macrophages, neutrophils) and structural cells (e.g., epithelial cells, fibroblasts) [5]. It serves as a key receptor mediating the biological effects of cytokines and corresponding chemokines. CXCR acts as a specific receptor for CXCLs with high affinity, forming a “receptor–ligand” biological axis; for instance, the CXCL12/CXCR4-7 regulatory axis serves as a pivotal molecular network in the initiation and progression of hematological malignancies, and the CXCL12/CXCR4 axis regulates the homing of leukemic cells to the bone marrow and their mobilization into the bloodstream through intricate interactions. Given that the root cause of hematological malignancies resides within the malignant stem cell niche, its cellular and secretory components, including CXCL12, play critical roles in regulating the survival, proliferation, and anchorage of leukemia stem cells, thereby contributing to drug resistance and disease recurrence [6,7,8]. Since acute myeloid leukemia (AML) and myelodysplastic syndrome (MDS) are associated with primordial-origin stem cells in the disease-initiating bone marrow microenvironment (BME), such stem cells cannot be eradicated through conventional therapies. The transcriptomic analysis of stem cell and progenitor cell populations in AML and MDS revealed that CXCL8 (IL-8) is one of the significantly overexpressed genes in patients with AML and MDS. Additionally, the CXCL8-specific receptor CXCR2 is overexpressed in samples from AML and MDS patients, as well as in several myeloid leukemia cell lines. Furthermore, CXCR2 has been identified as an adverse prognostic factor for MDS/AML, suggesting that the CXCL8/CXCR2 axis likely plays a crucial role in AML/MDS. The targeted inhibition of the CXCL8/CXCR2 axis represents a promising therapeutic strategy for MDS and AML [9,10]. The biological functions of the CXCR family and its specific ligands, the CXCLs, in hematological malignancies are summarized in Table 1.

**Figure 1:**
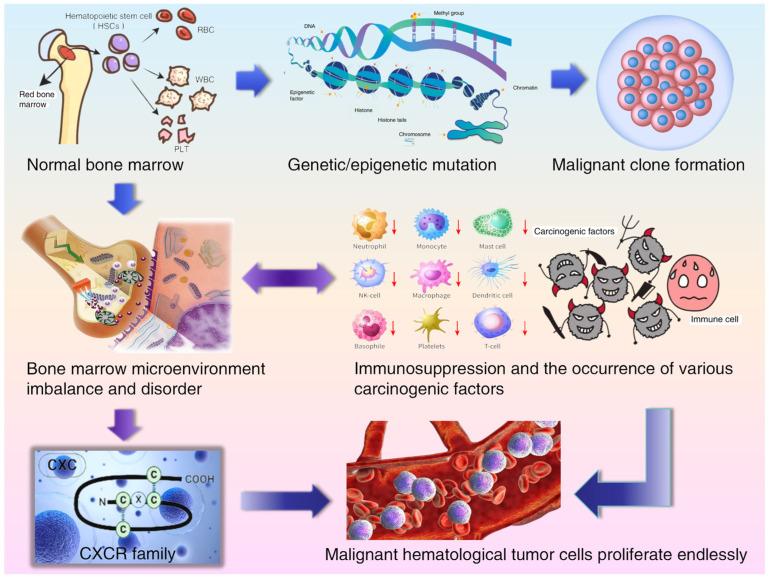
The occurrence and development mechanism of hematological malignancies in the bone marrow microenvironment.

The bone marrow (BM) serves as the primary hematopoietic organ in adults. It consists of blood vessels, nerve tissues, and diverse cell populations. These cells either directly contribute to blood cell production or support the hematopoietic function of tissues. All BM components work in concert to tightly regulate normal hematopoiesis, ensuring the generation of sufficient mature blood cells [84]. However, in various hematological malignancies, these physiological processes are disrupted and impaired, leading to reduced numbers of normal blood cells and immunosuppression. Considering the impact of hematological malignancy cells on the BME, the interaction between these malignancy cells and the BME can serve as a foundation for exploring the novel treatment strategies urgently required for these fatal diseases. Notably, within the BME, the chemotactic induction of CXCR family involves myeloid-derived suppressor cells (MDSCs), tumor-associated neutrophils (TANs), tumor-associated macrophages (TAMs), and regulatory T cells (Tregs), which perform various biological functions. These include maintaining homeostasis, inducing inflammatory responses, facilitating immune escape, promoting angiogenesis, regulating tumor cell biological effects, overcoming chemoresistance or immunoresistance, and repairing tissue damage [85]. The occurrence and development mechanism of hematological malignancies in the bone marrow microenvironment is shown in Figure 1. This paper aims to summarize, analyze, and discuss the roles and mechanisms of the CXCR family in hematologic malignancies, providing new references for both basic research and clinical practice in hematology.

## 2. CXCR Family and Myelodysplastic Syndrome (MDS)

MDS represents a highly heterogeneous myeloid malignancy caused by hematopoietic system dysplasia. It is characterized by low hematopoietic function, ineffective hematopoiesis, refractory pancytopenia, autoimmune abnormalities, and a high risk of leukemic transformation [86,87]. Its pathogenesis involves a complex molecular biology and cytogenetic abnormalities, including gene mutations and epigenetic changes. Additionally, the disorder of the immune microenvironment and cellular immune regulation mechanisms significantly contribute to MDS pathogenesis [88]. Exploring MDS pathogenesis and targeted therapies remains a challenging yet crucial focus in academic research.

Xiao et al. [89] demonstrated that in MDS patients with autoimmune diseases, the proportion of CD4^+^CXCR5^+^ cells in peripheral blood increases while programmed death receptor 1 (PD1) expression decreases; conversely, treatment reduces the proportion of CD4^+^CXCR5^+^ cells and increases PD1 expression. This suggests that the pro-inflammatory factor CXCR5 plays a vital role in MDS cell proliferation within the bone marrow microenvironment. Huselton et al. [90] revealed that combining the CXCL12/CXCR4 inhibitor DSTAT with azacytidine benefits MDS and refractory acute myeloid leukemia (AML) patients, indicating that CXCL12 and CXCR4, as the upregulated cell adhesion molecules for leukemia stem cells, enables the immune escape of blood tumor cells within the bone marrow microenvironment. The targeted inhibition of this regulatory axis may yield favorable outcomes for relapsed/refractory MDS and AML. Similarly, Schinke et al. [9] found that CXCL8-CXCR2 was overexpressed in the bone marrow cells of AML and MDS patients, with CXCR2 expression correlating with poor prognosis; after targeting and inhibiting the expression of CXCR2 via gene-editing technology and pharmacological means, the characteristics of stem cells in AML/MDS could be reduced, and the survival ability of cell lines could also be weakened, potentially serving as a therapeutic target for AML and MDS patients.

## 3. CXCR Family and Acute Myeloid Leukemia (AML)

AML is a series of hematologic tumors characterized by clonal hematopoietic stem/progenitor cell differentiation blockage and infinite malignant proliferation, representing the most common adult leukemia. Despite advancements in induced remission chemotherapy regimens, with hematopoietic stem cell transplantation, immunotherapy, or targeted therapy, only a minority of AML patients achieve remission, while the majority face relapse, refractoriness, or death, underscoring the status of AML as a major malignant disease threatening human life and health [91,92].

Previous studies [93,94] found that the interaction between leukemia cells and the bone marrow hematopoietic microenvironment is a critical cause of chemotherapy resistance and disease recurrence, with the CXCL12/CXCR4 axis playing a pivotal role in this interaction. A prior study by our team [95] revealed abnormally high CXCR1 and CXCR2 expression in acute leukemia (AL) patients, with no statistical difference in the relative expression levels between AML and ALL groups. The abnormal upregulation of CXCR1 and CXCR2 correlates with adverse clinical characteristics and laboratory indicators in AL patients, predisposing them to extramedullary infiltration, difficult-to-treat recurrence, and other poor prognostic outcomes, highlighting their importance in disease monitoring and prognosis evaluation. Intriguingly, Tang et al. [10] similarly demonstrated aberrantly high CXCR2 expression in AML patients, linking it to adverse clinical features and extramedullary infiltration, identifying it as an independent risk factor for poor patient prognosis.

Notably, CXCR4 is the most extensively studied chemokine receptor in malignant hematologic diseases, being closely associated with malignant proliferation, apoptosis regulation, chemotherapy resistance, and the bone marrow microenvironment of leukemia cells [96]. Both CXCR4 and its ligand CXCL12 are highly expressed in AML patients, correlating with adverse clinical features and laboratory indicators. Abnormally high CXCR4 expression serves as a critical marker of poor prognosis in AML. Targeting CXCR4 effectively mobilizes AML cells to the peripheral blood, prevents extramedullary organ infiltration and metastasis, and exhibits significant anti-leukemia efficacy with minimal toxic side effects, suggesting its potential as a personalized therapeutic target for AML [97,98].

Simultaneously, Lu et al. [13] analyzed the relationship between CXCR family members and AML using bioinformatics technology (TCGA), revealing that CXCR transcriptional expression plays a key role in AML pathogenesis and poor prognosis, with differential expression across AML subtypes. Correlations also exist between CXCR transcript expression and cytogenetic/molecular biological abnormalities, positioning the CXCR family as an important molecular marker for AML prognosis assessment. Hou et al. [99] further identified and screened the CXCRs that are valuable in AML diagnosis and prognosis via bioinformatics technology, conducting functional similarity analysis, immunoanalysis, enrichment analysis and drug prediction analysis, and constructing a prognosis survival model. Their findings confirmed that CXCR3, CXCR2, and CXCR6 are prognostic genes related to AML and its tumor immune microenvironment, offering new insights into AML prevention and treatment.

Cytarabine (Ara-C), as the most commonly used chemotherapy drug for the clinical treatment of AML patients, has been clinically applied in the field of hematology for decades. However, its drug resistance and toxic and side effects after increasing the dose have always been difficulties and challenges in clinical practice. To this end, actively seeking and utilizing novel targeted inhibitors of the CXCR family genes, and combining them with Ara-C treatment, may benefit AML patients. Noticeably, early investigations from our team utilized the CXCR1/2 small-molecule targeted allosteric inhibitor Reparixin, either alone or combined with Ara-C, to intervene in AML cells and explore its mechanism of action on malignant biological behaviors and influence on CXCR family expression; the results indicated that Reparixin combined with low-concentration Ara-C synergistically inhibits AML cell proliferation, invasion, migration, and clonal formation, inducing autophagy and apoptosis pathways. The molecular mechanism may involve modulating Bcl-2 family protein expression, downregulating CXCR family member expression, and inhibiting PI3K/AKT/NF-κB signaling pathway activation [100]. Interestingly, Verachi et al. [101] discovered that the CXCR1/2 targeted allosteric inhibitor Reparixin also improves myelofibrosis progression and serves as the first-line clinical treatment for myelofibrosis patients.

As previously discussed, the CXCL12/CXCR4 regulatory axis plays a critical role in homing and retention within the protective bone marrow microenvironment for hematologic tumor cells and normal hematopoietic stem cells (HSCs). Utilizing targeted CXCR4 inhibitors mobilizes hematologic tumor cells from the bone marrow into peripheral circulation, enabling effective eradication by chemotherapy agents, small-molecule inhibitors, or demethylation agents. A study indicated that drug-resistant leukemia strains remain unaffected by CXCR4 antagonists, whereas primary leukemia cells localize in bone marrow niches via surface CXCR4/CXCL12 interactions. Plerixafor, as a targeted CXCR4 inhibitor (FDA-approved), mobilizes leukemia cells post-hematopoietic cell transplantation, sensitizing them to cytotoxic therapy and enhancing healthy donor stem cell engraftment [102]. This highlights the potential value of CXCR4 antagonists as chemical sensitizers in preconditioning regimens and immune sensitizers for graft-versus-leukemia effects in allogeneic hematopoietic stem cell transplantation.

## 4. CXCR Family and Acute Lymphoblastic Leukemia (ALL)

Acute lymphoblastic leukemia (ALL) is a hematologic malignancy characterized by the uncontrolled proliferation of immature lymphoid cells. ALL is the most prevalent hematologic tumor in children and one of the most common forms of acute leukemia in adults, accounting for about 20–30% of adult AL, with a complete response rate (CR) of 70–90% and a 3–5-year disease-free survival rate (DFS) of 30–60% [103].

Despite significant advances in understanding the pathogenesis of ALL over the past few decades, leading to improvements in diagnosis, treatment, and prognostic monitoring, as well as advancements in genomics that have deepened our understanding of its molecular biology and genetic characteristics, targeted therapies such as tyrosine kinase inhibitors (TKIs) and immunotherapies like chimeric antigen receptor T cells (CAR-T) have been developed, offering hope for ALL patients [104]. However, due to the extensive clinical and biological heterogeneity of ALL, a considerable number of patients face treatment failure and the risk of relapse or refractory disease. Therefore, actively screening for new prognostic markers for treatment stratification and prognosis assessment remains of great practical significance, with the aim of further improving personalized diagnosis and treatment strategies for relapse and refractory ALL.

Zanetti et al. [105] demonstrated that high CXCR5 expression in pediatric B-ALL predicts a high risk of central nervous system recurrence. Tsaouli et al. [106] found that the Notch/CXCR4 signaling axis is involved in the pathogenesis and chemotherapy resistance of ALL, serving as an important molecular marker for disease progression. More importantly, high CXCR4 cell surface expression is a common hallmark of malignant proliferation and invasion, and mediates the generation of drug resistance and disease progression in B-cell acute lymphoblastic leukemia (B-ALL) and T-cell lymphoblastic leukemia (T-ALL); in addition, targeting Notch/CXCR4 therapy may provide beneficial therapeutic effects for ALL patients. Furthermore, relevant studies [34,107] have confirmed that CXCR4 and its ligands are highly expressed in ALL and closely associated with central nervous system invasion, recurrence, and poor prognosis. Targeting CXCR4 can effectively inhibit the malignant biological behavior of ALL, sensitize it to chemotherapy, prolong survival, and improve prognosis. Similarly, Philadelphia chromosome-positive acute lymphoblastic leukemia (Ph^+^ALL), characterized by chromosomal translocations between chromosomes 9 and 22, resulting in the expression of an oncogenic BCR-ABL1 fusion protein, relies on CXCR4 for the survival of BCR-ABL1-transformed mouse preB cells. The CXCR4 inhibitor JM#170, based on endogenous peptides, can effectively induce apoptosis in BCR-ABL1-transformed mouse B cells by activating the endogenous apoptotic pathway through the upregulation of c-Jun, Bim, and Bax genes, demonstrating potential as a novel targeted drug against Ph^+^ ALL [108].

Of concern, glucocorticoid (GC) resistance in recurrent B-cell acute lymphoblastic leukemia (B-ALL) in children poses a significant clinical challenge, with underlying mechanisms remaining poorly understood. In B-ALL, GC can induce drug resistance by abnormally activating the phospholipase C (PLC)-mediated cell survival pathway via CXCR4. Treatment with CXCR4 antagonists or PLC inhibitors improves the survival rate of dexamethasone (Dex)-treated NSG mice, indicating that the targeted inhibition of the CXCR4/PLC axis can significantly reverse the malignant proliferation of B-ALL cells and Dex resistance in ALL [109]. It should be noted that recent studies have shown that CXCR4 plays a critical role in maintaining and developing ALL by regulating and mediating upstream and downstream signaling molecules involved in disease pathogenesis. Specific inhibitors targeting CXCR4 can effectively reduce the infiltration and metastasis of ALL cells while weakening the intrinsic mechanisms of chemotherapy resistance [110,111,112].

Additionally, Shi et al. [71] used flow cytometry, scRNA-seq, and co-culture techniques to explore the in vitro toxicity of CXCR6^+^CD4^+^T cells induced by tumor antigens and peripheral blood mononuclear cells (PBMCs). Based on cell markers identified through in vivo scRNA-seq data, TARGET-ALL-P2 datasets, and integrated machine learning algorithms, ssGSEA was employed to identify key cells with prognostic value and simulate adoptive cell transfer therapy (ACT), and the study demonstrated that using CXCR6^+^CD4^+^T cells to mimic ACT therapy could reshape the bone marrow microenvironment to achieve anti-tumor effects, with CXCR6 not being a marker of resident memory CD4^+^T cells based on the expression of genes involved in their formation. Furthermore, therapeutic subtypes of pediatric B-ALL were defined for CD4^+^ cytotoxic T cell lineages. Simultaneously, Yang et al. [75] showed that miR-101 expression in T-ALL is negatively correlated with CXCR7 levels, a direct target of miR-101. Targeting the CXCR7/STAT3 axis via miR-101 can weaken the proliferation and metastasis of T-ALL cells. In conclusion, the CXCR family can be highly expressed in ALL, with complex signaling pathways among them. Targeting CXCR in the bone marrow microenvironment enhances the efficacy of traditional chemotherapy and synergistically inhibits ALL progression, providing a theoretical basis for new targeted therapies.

## 5. CXCR Family and Chronic Myelogenous Leukemia (CML)

Chronic myeloid leukemia (CML) is a blood tumor driven by the BCR-ABL fusion gene. The protein encoded by BCR-ABL is a non-receptor tyrosine kinase with uncontrolled activity in homologous or heterodimer forms. This results in the abnormal phosphorylation of tyrosine residues of various substrate proteins, activating multiple signaling pathways abnormally, ultimately leading to the malignant transformation and clonal proliferation of cells, and maintaining the disorder and suppression of apoptosis regulatory mechanisms, culminating in the occurrence and development of CML. Tyrosine kinase inhibitors (TKIs) effectively treat Philadelphia chromosome (Ph)-positive CML; however, TKI resistance remains the primary cause of treatment failure in clinical practice [113,114].

Kim et al. [115] demonstrated that Ponatinib, a third-generation tyrosine kinase inhibitor (TKI), was initially developed for the treatment of chronic myeloid leukemia (CML) patients harboring the T315I mutation. However, drug resistance or intolerance may lead to treatment interruption in CML patients. Furthermore, TKIs exhibit significant limitations in eradicating quiescent CML stem cells. It has been revealed that CXCR2 signaling is abnormally activated in response to chemotherapy-induced stress, and the CXCR2 antagonist SB225002 effectively inhibits Ponatinib-resistant CML cell proliferation, induces apoptosis, accumulates reactive oxygen species, and disrupts mitochondrial function, all of which are related to TKI chemotherapy resistance and apoptosis. Additionally, abnormally activated CXCR2 expression induces dipeptidyl peptidase IV (DPP4/CD26), a marker of CML leukemia stem cells, and inhibits the PI3K/Akt/mTOR pathway cascade. The targeted inhibition of CXCR2 eliminates CML stem cells and overcomes Ponatinib intolerance via the PI3K/Akt/mTOR and CD26 pathways. In conclusion, the targeted inhibition of CXCR2 holds promise as a viable therapeutic strategy for CML. It has also been shown that CXCR2 is highly expressed in the bone marrow of newly diagnosed CML patients and TKI-resistant CML cells. The CXCR2 antagonist SB225002 stalls CML cells in the G2/M phase, inhibiting proliferation while reducing mTOR, c-Myc, and BCR-ABL expression. SB225002 has been confirmed to inhibit tumor growth and significantly suppress TKI-resistant CML cells in mouse xenotransplantation models, indicating that CXCR2-targeted inhibitors represent a promising new therapeutic strategy for CML and TKI-resistant CML [22].

Notably, CML patients often harbor minimal or molecular residual disease (MRD), specifically leukemia stem cells (LSCs), which, despite being few in number, possess strong self-renewal and malignant proliferation potential, playing a key role in disease initiation, progression, recurrence, and drug resistance. However, LSCs predominantly reside in the G0 phase of the cell cycle, rendering them largely insensitive to chemotherapy drugs and capable of escaping therapeutic killing effects, conferring a strong survival advantage. Due to the unique nature of CML, disease recurrence is often caused by LSC dissemination, and TKIs cannot eradicate LSCs. Agarwal et al. [116] analyzed the changes in bone marrow mesenchymal cell populations induced by leukemia using mouse CML models, revealing that tumor necrosis factor α (TNF-α) enhances LSC maintenance and growth by mediating the CXCL1-CXCR2 signaling axis in the CML bone marrow microenvironment. This highlights the importance and potential of inflammatory signals in expanding and modifying bone marrow stromal cells (BMSCs) in CML patients via TNF-α, although based on murine models. Specifically, TNF-α mediates increased CXCL1 expression in CML BMSCs through signal transduction pathways, interacting with highly expressed CXCR2 in LSCs, thereby inducing a CXCR2 selection-dependent mechanism leading to the unlimited expansion of LSCs and leukemia progenitor cells. Combining CXCR2 antagonist with TKIs effectively inhibits LSC proliferation in CML, significantly enhancing the inhibitory effect on leukemia cells and LSCs while promoting CML cell apoptosis. This confirms the critical role of paracrine CXCL1-CXCR2 signaling in supporting LSC growth and TKI resistance.

Moreover, Herrmann et al. [117] demonstrated that CXCR4 is overexpressed in CML, and LASP1 has been proven to effectively bind to CXCR4, which is associated with the recurrence mechanism of CML. Knocking down LASP1 levels combined with the high expression of CXCR4 could help weaken TKI resistance. Meanwhile, Li et al. [79] confirmed that the co-culture of the CML cell lines K562 and KU812 with bone marrow stromal cells M2-10B4 could weaken imatinib (IM)-induced apoptosis. The CXCL12/CXCR7 pathway was activated in the CML co-culture model. Silencing CXCR7 was linked to TKI resistance, and when CXCR7 was activated, extracellular signal-regulated kinase (ERK) phosphorylation occurred, activating the expression of downstream anti-apoptotic proteins. The use of flavonoids such as oroxylin A can effectively reverse IM resistance in CML within the bone marrow microenvironment by enhancing the sensitivity of CML cells to IM therapy through the regulation of the CXCL12/CXCR7 pathway.

## 6. CXCR Family and Chronic Lymphocytic Leukemia (CLL), Malignant Lymphoma (ML)

Chronic lymphocytic leukemia (CLL), also known as chronic lymphocytic leukemia/small lymphocytic lymphoma (CLL/SLL), is the most common type of leukemia in adults in Western countries. It is a clonal proliferative tumor of mature B lymphocytes, prevalent and still incurable in an aging society. With the increasing aging population around the world, the number of CLL patients is also showing an upward trend annually [118]. CLL is often characterized by the aggregation and involvement of CD5 and CD23-positive, mature monoclonal lymphocytes in the bone marrow, peripheral blood, lymph nodes, spleen, and other tissues and organs. The prognosis of CLL patients is highly heterogeneous and closely related to age, clinical stage, cytogenetics, and molecular biological indicators [119,120]. Meanwhile, lymphoma is the most common malignant solid tumor of the hematological system. Due to its high heterogeneity, lymphoma can occur in various parts of the body and may also manifest as leukemia (such as the subtype of lymphoblastic lymphoma). The disease is typically characterized by non-specific symptoms or signs such as painless progressive lymph node enlargement, fever, wasting, fatigue, pruritus, and night sweats. Lymphoma can invade extranodal tissues and organs such as the nasopharynx, gastrointestinal tract, bones, and skin, causing involvement and damage to corresponding organs. Currently, the diagnosis of lymphoma relies on pathological biopsy for immunohistochemical and related molecular biological detection. The histopathological types of lymphoma include Hodgkin lymphoma (HL) and non-Hodgkin lymphoma (NHL). In clinical practice, the treatment of lymphoma primarily involves a comprehensive treatment regimen based on chemotherapy [121,122,123].

In the study of host–human T-cell leukemia virus type 1 (HTLV-1)-associated adult T-cell leukemia/lymphoma (ATLL), Rahimzada et al. [124] found that compared with asymptomatic carriers of HTLV-1 (ACs), CXCR3 expression was significantly decreased in ATLL patients. The mean HTLV-1 proviral load (PVL) in ATLL patients was higher than in ACs, and Caspase-1 showed significant differences between ATLL and ACs. This suggests that CXCR3 likely participates in the early development of ATLL into acute lymphoma, and that the mechanism may involve regulating pyroptosis. As chemotaxis is an important biological behavior process often used by tumor cells for metastasis and invasion, as mentioned above, CXCR4, its ligand CXCL12, and atypical receptor ACKR3 are overexpressed in many human cancers. Antonello et al. [125] found that LTB4 acted synergistically with CXCL12 to stimulate the migration of VAL cells in diffuse large B-cell lymphoma. The inhibition of ACKR3 expression via pharmacological or molecular genetic techniques could impair the migration of lymphoma cells toward CXCL12 and significantly reduce chemotaxis to CXCL12. This further demonstrated that ACKR3 enhances CXCL12/CXCR4-mediated intercellular-induced lymphoma migration by promoting LTB4 production, revealing the cell-to-cell-induced migration of lymphoma. Of concern, the concurrent “tumor sink effect” in solid tumors is a novel and interesting direction of study, referring to the reduced absorption of normal organs in patients with a high tumor load; however, this phenomenon has not been applied to evaluate radiotracers for blood tumors. Kosmala et al. [126] used CXCR4[^68^Ga]Ga-PentixaFor PET/CT imaging to detect the potential “lymphoma sink effect” in patients with marginal zone lymphoma (MZL), but unfortunately, this study showed no association between normal organ uptake and the CXCR4-positive lymphoma burden in MZL patients, suggesting that the lymphoma sink effect of MZL may not be significant relative to other therapeutic radiotracers used for the imaging and treatment of solid tumors. This finding may be related to the therapeutic setting. The missing lymphoma sink effect may also be related to dosing studies using novel CXCR4 inhibitors as “cold” drugs.

At the same time, Nugent et al. [127] found that some lymphoblastic lymphomas express CXCR3, while CXCR3, CXCR4, and CXCR5 are significantly highly expressed in marginal zone lymphomas. Negative CXCR3 in immunohistochemical staining is a unique subtype of marginal zone lymphoma, and there is a significant correlation between the CXCR4 expression level and bone marrow involvement. Meanwhile, most mantle cell lymphomas express CXCR4 and CXCR5. CXCR5, originally named Burkitt lymphoma receptor 1 (BLR1), is upregulated in Burkitt lymphoma. The study also found that CXCR3-6 was highly expressed in follicular lymphoma and diffuse large B-cell lymphoma, and was associated with poor prognosis. Lymphoplasmacytic lymphoma (LPL) and Waldenström macroglobulinemia (WM) are rare indolent lymphomas caused by fully differentiated B cells. A related study [128] showed that CXCR4 is highly expressed in LPL/WM patients and shows significantly higher BM disease involvement, serum immunoglobulin-M levels, and symptomatic disease requiring therapy, and is also regarded as a risk factor for BM infiltration. Driver mutations in CXCR4 are important determinants of the clinical phenotype and survival in LPL/WM patients. Currently, the role of CXCR4 in the homing, migration, and infiltration of LPL/WM cells has been revealed. The intervention of WM cell lines with CXCR4 antagonists inhibited migration and signal transduction.

In addition, Lewis et al. [129], by preparing and using an acquired CXCR4 mutant mouse model with the overactivation of CXCR4 signal transduction, confirmed that CXCR4 overactivation would lead to disease progression and a more aggressive phenotype in the mouse Eμ-TCL1 CLL model. Moreover, MYC activation in invasive lymphoma is associated with an increased expression of CXCR4. Overactivated CXCR4 signaling synergistically induces a unique oncogenic transcriptional program in B cells through the PLK1/FOXM1 pathway mediated by TCL1, suggesting that abnormal CXCR4 activity is associated with pathogenesis, disease progression, and resistance to chemotherapy in malignant lymphoma. CXCR4 overactivation is a co-driver of the aggressive lymphoma phenotype. Another study [130] confirmed that the CXCR4-targeted inhibitor AMD11070 and its derivatives could induce the expression of pro-apoptotic genes from the Bcl-2 family in CXCR4-positive lymphoma cell lines while downregulating the expression levels of Jun N-terminal kinase (JNK), ERK1/2, and nuclear factor kappa-B (NF-κB); it suggested that the CXCL12-CXCR4 axis mediates the pathogenesis of diffuse large B-cell lymphoma (DLBCL), which represents a potential therapeutic target for aggressive lymphomas. Cui et al. [74] found that the plasma tissue factor pathway inhibitor (TFPI) level in CLL patients was higher than in the healthy control group, especially in advanced CLL patients. TFPI could enhance the CXCL12-mediated transendothelial migration (TEM) of CLL cells by upregulating the expression of the CXCL12 receptor CXCR7 in the in vitro study, but not CXCR4; they also found that these biological effects could be eliminated by the CXCR7 inhibitor CCX771.

## 7. CXCR Family and Multiple Myeloma (MM)

As a malignant proliferative disease derived from monoclonal plasma cells, MM produces a large amount of monoclonal immunoglobulin in patients, causing the irreversible loss and severe damage of end-stage organ functions, including bone marrow failure, renal failure, heart failure, refractory infection and hypercalcemic crisis [131,132]. It is a common and incurable malignant hematologic disease in middle-aged and elderly people. In recent years, the incidence of MM has shown an increasing trend year by year, and it is currently the second most common hematologic tumor after NHL. Since MM is still a malignant and incurable hematologic disease with an unclear etiology and mechanism, chemotherapy resistance is the main challenge associated with MM treatment failure and difficult-to-treat recurrence [133,134,135].

Studies [52,136] have confirmed that the CXCR4/CXCL12 axis plays a particularly critical role in MM cell proliferation, invasion, and chemoresistance; in addition, CXCR4 also plays a pleiotropic role in the expansion and colonization of MM cells in the BM and in the homing, adhesion, invasion, migration, and mobilization of MM cells out of the BM. An abnormally high expression of CXCR4 can interact with various molecular signaling pathways, participate in osteoclast generation in MM, and mediate the malignant proliferation of MM cells. Targeting the CXCR4 axis and novel combination therapies may be a new effective strategy for treating MM. It has also been shown that the high expression of CXCL12/CXCR4 in the bone marrow of MM patients is closely related to the occurrence and progression of MM diseases, and a high expression of CXCL12/CXCR4 predicts poor prognosis of MM patients [137]. Correspondingly, Bonanni et al. [27] found that CXCR3 inhibits the anti-tumor ability of NK cells in vivo, and targeting CXCR3 can increase the bone marrow homing ability of IL-15. This effect is related to an increase in the bone marrow homing of NK cells in vivo, thereby exerting scavenging ability against MM cells. Antagonizing CXCR3 will help exert an anti-MM function on NK cells in the bone marrow and improve the efficacy of the adoptive immunotherapy of MM based on NK cells. In addition, CXCR4 expression is abnormally elevated in MM extramedullary infiltration and plasma cell tumors [138], and blocking the CXCL12/CXCR4 axis could reduce its interaction with IL-6, decrease adhesion-mediated chemoresistance in MM cells, and induce cell apoptosis; mechanistically, SDF-1/CXCR4 may up-regulate the expression of IL-6 through the activation of the P13K/AKT signaling pathway, thereby affecting the chemoresistance mediated by adhesion in MM cells [139]. At the same time, relevant studies [140,141] also showed that the CXCR4-targeted inhibitor Plerixafor combined with bortezomib could be used as a chemotherapy sensitization strategy for relapsed/refractory MM, with good safety and a high objective remission rate. Moreover, Plerixafor could also be applied to the mobilization of autologous transplantation hematopoietic stem cells in MM patients, with good efficacy and safety.

## 8. Summary and Prospect

**Figure 2:**
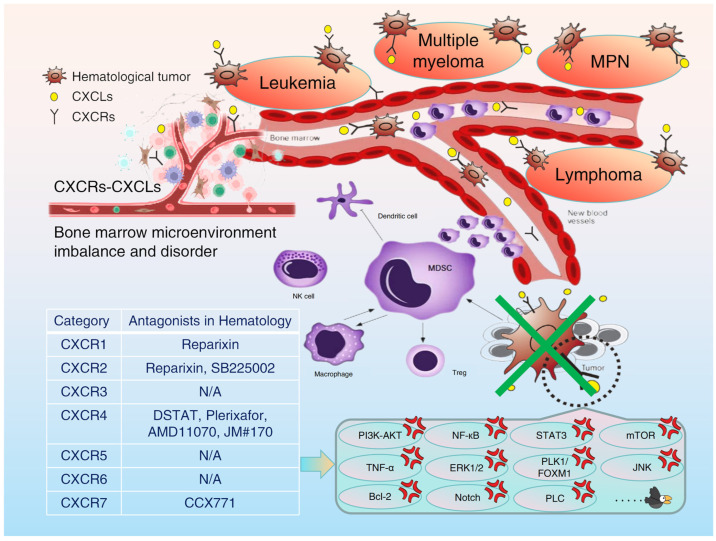
Potential therapeutic value and significance of antagonizing the CXCR family in the bone marrow microenvironment for hematological malignancies.

In the bone marrow microenvironment, various chemokine imbalances play an important role in promoting tumor occurrence, progression, chemoresistance, and immune escape. Due to the drug resistance or poor efficacy of conventional chemotherapy monotherapy in malignant hematologic diseases, most patients still face the painful reality of relapse and difficult treatment. As mentioned above, the CXCR family is abnormally expressed in many malignant hematologic diseases and plays an indispensable role in the malignant biological behaviors of tumors through various complex molecular biological regulation mechanisms. For this reason, Table 2 lists and summarizes the clinical significance and prognostic value of the CXCR family in patients with hematologic malignancies; this has attracted extensive attention from the academic community. Accordingly, a combination of the targeted CXCR family or CXCL/CXCR biological axis and chemotherapy drugs can achieve a good anti-tumor effect; a further understanding of the CXCR family and its regulatory network in the bone marrow microenvironment will provide promising molecular markers for clinical decisions, basic or clinical research such as diagnosis, treatment, and the prognosis evaluation of malignant hematologic diseases (Figure 2). Correspondingly, research on the application of CXCR family-specific targeted inhibitors (antagonists) in hematological malignancies is detailed in Table 3.

## 9. Conclusions

In conclusion, this paper summarizes and discusses the mechanism of action and the latest progress in targeted therapy using the CXCR family and its ligand CXCL in malignant hematologic diseases. It is believed that the regulatory role and molecular mechanism of the CXCR family in the BME of hematologic malignancies is an active area of investigation and is expected to be further elucidated. In addition, the development and clinical application of new targeted inhibitors, immunotherapy, or cell therapy targeting the CXCR family are likely to open up new prospects for the field of hematology.

## Figures and Tables

**Table 1:** The biological functions and involved signal pathways of the CXCR family in hematologic malignancies.

Category	Specific Ligands	Biological Function in Hematological Malignancies	Involved Signal Pathways	References
CXCR1	CXCL3, CXCL5, CXCL6, CXCL8	Regulate processes such as the functionality of leukemia stem cells (LSCs), tumor microenvironment interactions, and drug resistance mechanisms	PI3K/AKT/mTOR; JAK/STAT3; MAPK/ERK; NF-κB	[11,12,13,14]
		Regulate the proliferation and invasion of lymphoma cells within the tumor microenvironment	NF-κB	[15]
		Mediate the progression of myeloma by influencing cellular signaling pathways	NF-κB	[16]
		Promote occurrence and development of primary myelofibrosis	JAK2/STAT/MAPK; TPO/MPL/JAK2;AKT/NF-κB	[17,18,19]
CXCR2	CXCL1, CXCL2, CXCL3, CXCL5, CXCL6, CXCL7, CXCL8	Promote occurrence and development of primary myelofibrosis	JAK2/STAT/MAPK; TPO/MPL/JAK2; AKT/NF-κB	[17,18,19]
		Promote the survival and the generation of chemotherapy resistance of AML cells	NF-κB2/MIF	[20,21]
		Inhibit the proliferation of CML cells, promote apoptosis, and overcome drug resistance	AKT/mTOR/c-Myc	[22]
		Promote the proliferation of MM cells, activate osteoclast signaling pathways, and mediate disease progression	JAK/STAT3	[23]
		Facilitate the development of myeloproliferative neoplasms, including myelofibrosis	JAK/STAT/NF-κB	[24]
CXCR3	CXCL4, CXCL9, CXCL10, CXCL11, CXCL13	Enhance the malignant biological characteristics of mantle cell lymphoma (MCL) cells, including proliferation, migration, invasion, and inhibition of apoptosis	JAK1/STAT1; β-catenin/c-Myc	[25]
		Promote the malignant proliferation and immune dysfunction associated with Epstein–Barr virus (EBV)-positive lymphomas	NF-κB/p38 MAPK	[26]
		Enhance the efficacy of adoptive metastasized activated natural killer (NK) cells against multiple myeloma	IL-12/15/18	[27]
CXCR4	CXCL3, CXCL12	Promote the survival, proliferation, migration, and drug resistance of AML cells	PI3K/AKT/mTOR; MAPK/ERK; Wnt/β-catenin; VLA-4/CAM-DR	[28,29,30,31,32]
		Regulate the migration, homing, survival, and drug resistance of ALL cells, thereby affecting disease progression and treatment response	PI3K/AKT/mTOR; JAK/STAT; Rac1/RhoA	[33,34,35]
		Participate in the microenvironmental dependence, cell migration, survival, and drug resistance of CLL cells	JAK/STAT; VLA-4/CAM-DR; NF-κB; PI3K/AKT/mTOR; MAPK/ERK; Rac1/RhoA	[36,37,38,39,40,41,42]
		Maintain the self-renewal capacity of CML LSCs, bone marrow microenvironment dependence, drug resistance, and disease progression	PI3K/AKT/mTOR; MAPK/ERK; JAK/STAT; NF-κB; Wnt/β-catenin	[43,44,45,46,47,48]
		Regulate the interaction between the bone marrow microenvironment, tumor cell homing, drug resistance, and disease progression in patients with MM	PI3K/AKT/mTOR; MAPK/ERK; NF-κB; JAK/STAT3; RANK/RANKL/OPG	[49,50,51,52,53]
		Regulate lymphoma cell migration, microenvironmental dependence, drug resistance, and immune escape to promote disease progression	PI3K/AKT/mTOR; MAPK/ERK; JAK/STAT; NF-κB	[54,55,56,57,58,59,60,61]
CXCR5	CXCL13	Regulate the proliferation, migration, survival of leukemia cells, and their interaction with the microenvironment to promote disease progression	MAPK/ERK JAK/STAT VLA-4; CAM-DR	[62,63,64,65,66]
		Regulate the proliferation, migration, survival of lymphoma cells, and their interaction with the immune microenvironment to promote tumor progression	NF-κB; VLA-4	[66,67,68]
		Mediate the regulation of the tumor microenvironment (TME), cell proliferation, migration, and the development of drug resistance in multiple myeloma	NF-κB; p53	[69,70]
CXCR6	CXCL12, CXCL16	Mediate the immune escape, proliferation, and interaction with the BME of leukemia cells	Not yet mentioned	[71]
		Regulate the tumor microenvironment of lymphoma, promote cell migration, and enhance survival signals	death receptor-caspase, TNF, NF-κB	[72]
CXCR7	CXCL12	Participate in the maintenance of LSCs, bone marrow microenvironment interactions, and drug resistance	Wnt/β-catenin; STAT3; ERK/MAPK	[73,74,75,76,77,78,79]
		Mediate the progression of lymphoma, microenvironmental adaptation, and drug resistance	CXCL12-CXCR4	[80,81]
		Play a critical role in bone marrow microenvironment colonization, treatment resistance, and disease progression in multiple myeloma	CAM-DR; PI3K/AKT; β-arrestin; ERK	[82,83]
CXCR8 (GPR35)	CXCL17	Not yet clearly reported	Not yet clearly reported	-

**Table 2:** The prognostic value of the CXCR family in patients with hematologic malignancies.

Category	Prognostic Value for Different Hematological Malignancies	Country	Number of Patients Included	References
CXCR1	High expression of CXCR1/2 in AL indicates a poor prognosis and a low long-term survival rate	China	86 cases	[95]
	A diagnostic marker for aggressive NK cell leukemia (ANKL)	Japan	15 cases	[142]
	A diagnostic marker for T-cell large granular lymphoblastic leukemia	Portugal	46 cases	[143]
	A molecular marker for the progression of MM	Italy	44 cases	[144]
	As a diagnostic marker for patients with MPN	Italy	9 cases	[145]
	A therapeutic target for myelofibrosis	France	37 cases	[19]
CXCR2	High expression of CXCR1/2 in AL indicates a poor prognosis and a low long-term survival rate	China	86 cases	[95]
	High expression of CXCR2 in AML is an independent risk factor for poor prognosis	China	83 cases	[10]
	As one of the co-diagnostic markers for patients with CLL	Greece	62 cases	[146]
	A diagnostic marker for T-cell lymphoma	Netherlands	17 cases	[147]
CXCR3	An evaluation marker for the diagnosis and prognosis of CLL	Czech Republic	60 cases	[148]
	A molecular marker for the diagnosis and differentiation of CLL/SLL	UK.	78 cases	[149]
	A diagnostic marker and therapeutic target for mantle cell lymphoma (MCL)	China	30 cases	[25]
	A diagnostic marker for angioimmunoblastic T-cell lymphomas (AITLs)	Korea	82 cases	[150]
	An indicator for evaluating the adverse clinical characteristics and prognosis of extranodal lymphoma	Egypt	78 cases	[151]
	It is related to the survival rate of patients with primary central nervous system lymphoma	Japan	31 cases	[152]
	A molecular marker for distant dissemination and disease progression of MM	Italy	20 cases	[153]
CXCR4	High expression of CXCR4 is an independent prognostic factor for recurrence and poor OS in AML patients	China	134 cases	[154]
	A marker of poor prognosis in AML patients	Egypt	58 cases	[155]
	A marker of extramedullary invasion and poor prognosis in children with ALL	Austria	73 cases	[156]
	CXCR4 is abnormally highly expressed in both childhood and adult ALL and is associated with shorter disease-free survival and overall survival	South Korea	54 cases	[157]
	High expression of CXCR4 is associated with poor prognosis and is an independent predictor of survival in AML	China	122 cases	[158]
	High expression of CXCR4 is associated with poor prognosis and chemotherapy resistance in patients with CLL, and it is a key molecular marker for disease prediction	Egypt	30 cases	[159]
	High expression of CXCR4 is associated with poor clinical characteristics and poor laboratory indicators of lymphoma, and in germinal center B-cell-like (GCB)-DLBCL, CXCR4 is an independent factor for predicting poor PFS	China	743 cases	[160]
	High expression of CXCR4 is positively correlated with brain metastasis of DLBCL and is associated with poor overall survival rate, and it is an independent prognostic factor for DLBCL	China	61 cases	[161]
	The upregulation of CXCR4 expression in ABC-DLBCL suggests a poor prognosis	China	103 cases	[162]
	CXCR4 is an independent risk factor for poor prognosis of MCL and is an ideal target for therapy	Canada	146 cases	[163]
	The decreased expression of CXCR4 in the BM of NHL patients after chemotherapy suggests a good prognosis	Poland	26 cases	[164]
	CXCR4 is an independent predictor of survival prognosis for patients with MM	China	227 cases	[165]
	Low expression of CXCR4 indicates a poor prognosis in MM patients and a high incidence of extramedullary lesions	China	48 cases	[166]
	Low expression of CXCR4 indicates poor clinical characteristics and indicators as well as disease progression in patients with MPN	Italy	100 cases	[167]
	High expression of CXCR4 suggests a poor prognosis for patients with MDS	China	81 cases	[168]
CXCR5	CXCR5 is highly expressed in patients with MDS and is an indicator for therapeutic effect evaluation	China	42 cases	[89]
	Abnormally high expression of CXCR5 indicates the disease progression and poor prognosis of DLBCL	China	71 cases	[169]
	The CXCR5 polymorphism has a relatively low correlation with DLBCL and PTCL, and is not related to the risk of MCL or MZL. However, it has certain value for the diagnosis and prognosis of FL patients	USA	2694 cases	[170]
CXCR6	Not yet clearly reported	-	-	-
CXCR7	CXCR7 is highly expressed in acute monocytic leukemia and is a key molecular marker for disease prediction	China	10 cases	[171]
	CXCR7 is an independent prognostic factor for lymphoma and is associated with good clinical outcomes	Spain	94 cases	[172]
	CXCR7 is abnormally highly expressed in MM patients, which may be related to the disease progression	USA	20 cases	[83]
CXCR8 (GPR35)	Not yet clearly reported	-	-	-

**Table 3:** The research on the application of CXCR family-specific targeted inhibitors (antagonists) in hematological malignancies.

Category	Specific Inhibitors (Antagonists)	Application of the Types of Hematological Malignancies	Specific Therapeutic Effect	References
CXCR1	Reparixin	AML	Inhibits the malignant biological behaviors of AML cells and induce autophagy and apoptosis	[100]
	Reparixin	MPN (myelofibrosis)	Slows down the process of myelofibrosis	[145]
CXCR2	Reparixin	AML	Inhibits the malignant biological behaviors of AML cells and induce autophagy and apoptosis	[100]
	SB225002	CML	Overcomes TKI resistance in CML	[22]
		ALL	Induces the death of ALL cells and cell cycle arrest, and have an anti-leukemia effect	[173]
CXCR3	Not yet clearly reported	-	-	-
CXCR4	Plerixaflor (AMD3100)	AL, MDS	Improves the tolerance and remission rate of patients with relapsed/refractory acute leukemia and MDS	[174]
		MDS	Enhances the sensitivity of MDS patients to azacitidine and increase the remission rate	[175]
		CLL	Enhances the sensitivity and tolerance of chemotherapy drugs	[176]
		AML	A BM clearance preparation protocol for allogeneic transplantation in AML patients	[177]
		AML	The safety and efficacy of treating newly diagnosed elderly patients with acute myeloid leukemia are good	[178]
		ALL	A chemotherapy sensitizer for children with ALL	[179]
		ALL	Induces the long-term mobilization of mouse ALL cells, increase the proportion of circulating cells in the blood, enhance the efficacy of chemotherapy and improve survival	[180]
		CML	Ineffective in reducing the burden of leukemia, and infiltration of the central nervous system may occur, and the beneficial effect is limited when it is combined with TKIs	[181]
		CML	Enhances the sensitivity of BCR-ABL^+^ cells to imatinib and nilotinib	[182]
		CLL	Improves the efficacy of chemotherapy and enhance chemotherapy-induced sensitization	[183]
		R/R DLBCL	A salvage treatment method and improve the therapeutic effect	[184]
		PCNSL	Improves the therapeutic effect and control the progression of lymphoma	[185]
		MM	A bone-marrow-mobilizing agent for patients with MM to improve the success rate of bone marrow transplantation	[186,187,188,189,190]
	DSTAT (CX-01)	AML, MDS	Improves the therapeutic effect and the effective remission rate	[90]
		AML	Enhances the efficacy of chemotherapy and improve tolerance	[191]
	Olaptesed pegol (NOX-A12)	CLL	Improves the efficacy of chemotherapy and chemosensitization while enhancing the tolerance of patients	[192]
	Motixafortide	MM	Stem cell mobilization for MM	[193,194,195]
	Ulocuplumab (BMS-936564/MDX1338)	R/R MM	Chemotherapy sensitizes and improves therapeutic effects	[196]
		CLL	Induces the cell death of chronic lymphocytic leukemia through the reactive oxygen species dependent pathway	[197]
		ML, NHL, CLL	Monotherapy can show anti-tumor activity against AML, NHL and MM xenotransplantation models	[198]
CXCR5	Not yet clearly reported	-	-	-
CXCR6	Not yet clearly reported	-	-	-
CXCR7	CCX771	CLL	Inhibits the trans-endothelial migration (TEM) of CLL cells	[74]
CXCR8 (GPR35)	-	-	-	-

## Data Availability

No new data were created or analyzed in this study.

## Abbreviations

The following abbreviations are used in this manuscript:

CXCR	C-X-C motif chemokine receptor
CXCL	C-X-C motif chemokine ligand
BME	Bone marrow microenvironment
TME	Tumor microenvironment
MDS	Myelodysplastic syndrome
AL	Acute leukemia
AML	Acute Myeloid Leukemia
ALL	Acute lymphoblastic leukemia
CML	Chronic myeloid leukemia
CLL	Chronic lymphocytic leukemia
ML	Malignant lymphoma
CLL/SLL	Chronic lymphocytic leukemia/small lymphocytic lymphoma
MM	Multiple myeloma
MPN	Myeloproliferative neoplasm
HL	Hodgkin lymphoma
NHL	Non-Hodgkin lymphoma
MDSCs	Myeloid-derived suppressor cells
TANs	Tumor-associated neutrophils
TAMs	Tumor-associated macrophages
Tregs	Regulatory T cells
NK cells	Natural killer cells
PD1	Programmed death receptor 1
PI3K	Phosphatidylinositol 3-kinase
AKT	Protein kinase B
NF-κB	Nuclear factor kappa-B
HSCs	Hematopoietic stem cells
CR	Complete response
DFS	Disease-free survival rate
CAR-T	Chimeric antigen receptor T cells
PLC	Phospholipase C
PBMCs	Peripheral blood mononuclear cells
ACT	Adoptive cell transfer therapy
STAT3	Signal transducer and activator of transcription 3
TKIs	Tyrosine kinase inhibitors
Ph	Philadelphia chromosome
mTOR	Mammalian target of rapamycin
MRD	Minimal or molecular residual disease
LSCs	Leukemia stem cells
TNF-α	Tumor necrosis factor α
BMSCs	Bone marrow stromal cells
ERK	Extracellular signal-regulated kinase
HTLV-1	Host–human T-cell leukemia virus type 1
ATLL	Adult T-cell leukemia/lymphoma
PVL	Proviral load
MZL	Marginal zone lymphoma
BLR1	Burkitt lymphoma receptor 1
LPL	Lymphoplasmacytic lymphoma
WM	Waldenström macroglobulinemia
PLK1	Polo-like Kinase 1
FOXM1	Forkhead box protein M1
JNK	Jun N-terminal kinase
DLBCL	Diffuse large B-cell lymphoma
TEM	Transendothelial migration
